# Chronic hypoxia reshapes the brain: subregional volume reductions of the hippocampus and amygdala contrasts with behavioral resilience in long-term high-altitude migrants

**DOI:** 10.3389/fnins.2026.1789272

**Published:** 2026-03-26

**Authors:** Zhiwei Zuo, Xiaolong Chen, Jianguo Huang, Huilan Li, Guo Li, Jing Zhu, Peng Wang, Shusu Du, Jian Zhou, Feizhou Du

**Affiliations:** 1 Department of Radiology, General Hospital of Western Theater Command, Chengdu, China; 2 Department of Medical Engineering, The 945th Hospital of PLA, Ya’an, China; 3 Department of Radiology, The 945th Hospital of PLA, Ya’an, China; 4 Maintenance Technology Research Office, The 922326th Unit of PLA, Zhanjiang, China

**Keywords:** amygdala, brain volume, depression, high altitude, hippocampus

## Abstract

**Background:**

Individuals relocating from low- to high-altitude areas typically encounter serious health challenges due to harsh environmental conditions. Although individual adaptation varies, cognitive decline and mental health disturbances are common in these migrants. This study sought to comprehensively investigate the morphological changes of hippocampal and amygdalar subregions in long-term migrants transitioning from low to high altitudes.

**Methods:**

Forty-four young adult male Han Chinese low-to-high-altitude migrants (HAs) and 44 age-matched male low-altitude Han Chinese residents (LAs) underwent three-dimensional high-resolution structural magnetic resonance imaging and neuropsychological testing. Gray matter volume at both hemispheric and hippocampal/amygdalar subregional levels was calculated. The relationships between altered subregional volumes and neuropsychological performance in HAs were then analyzed.

**Results:**

Compared with that of the LA group, the gray matter volume of the HA group was basically maintained at the bilateral cerebral cortex and cerebellar cortex but significantly reduced in multiple hippocampal and amygdalar subregions. Moreover, longer residence at high altitude was associated with fewer insomnia symptoms. Additionally, volume decreases in specific hippocampal and amygdalar subregions in the HA group were significantly related to the severity of insomnia and the duration of high-altitude exposure.

**Conclusion:**

Our findings reveal complex patterns of gray matter alterations in long-term HAs. The correlations among specific morphological changes, duration of high-altitude residency, and insomnia measurements suggest a potential neuroadaptive mechanism.

## 1 Introduction

With the ongoing implementation of China’s Western Development Strategy and the steady development of high-altitude infrastructure, an increasing number of low-altitude residents are migrating to high-altitude areas, such as the Qinghai-Tibet Plateau. The low- to high-altitude migrants (HAs) include a diverse group of aid-Tibet cadres, infrastructure construction workers, military personnel, laborers, and business operators, most of whom are young Han Chinese males.

These HAs usually encounter a variety of health challenges due to the harsh high-altitude environment, which is characterized by hypoxia, low atmospheric pressure, large daily temperature fluctuations, strong ultraviolet radiation, etc. Acute mountain sickness (AMS), the most common type of high-altitude illness, primarily manifests as headache, nausea, and insomnia (Frank et al., 2022). Although many HAs can adapt to plateau life within a few months, some may experience chronic mountain sickness (CMS), such as high-altitude polycythemia or hypertension (Gao et al., 2020). Long-term hypoxic exposure may impair cognitive function, resulting in memory decline, attention deficits, and executive dysfunction (Li and Wang, 2022). Drastic changes in living conditions, as well as the pressures of social adaptation, can also lead to various psychological health issues such as mood swings, tension, irritability, depression, and anxiety, especially in individuals with a genetic predisposition (Quaresma et al., 2020). Due to physical factors, some HAs ultimately cannot tolerate prolonged exposure to high-altitude environments, which may force them to return to their original altitudes.

Our understanding of physiological compensation mechanisms and neural remodeling processes in a high-altitude environment was limited for ages. In recent years, with the development of neuroimaging techniques, knowledge of cerebral adaptive changes and their mechanisms related to the high-altitude environment has been enhanced. According to a multi-parameter neuroimaging study (Zhang et al., 2023), alteration patterns of gray matter volume and functional connectivity in HAs were similar to those in Tibetans, suggesting that the neuromodulatory mechanisms’ response to hypoxic stressors in different populations may be analogous. A recent diffusion tensor imaging (DTI) study (Liu et al., 2024) found that long-term HAs showed irreversible impairment in the corpus callosum and did not display any logical memory impairment. Because the corpus callosum is related to logical memory function (Liu et al., 2024), these results suggested potential adaptive neural compensations for long-term high-altitude exposure. However, the degree, reversibility, and individual differences in neurocognitive or mental changes in long-term HAs still need further systematic investigation.

Hypoxia plays a crucial role in brain development, aging, and the onset of age-related neurological disorders. The shared pathophysiological processes involved in the above-mentioned conditions include oxidative stress, neuroinflammation, mitochondrial dysfunction, etc. (Burtscher et al., 2021), to which the hippocampus and amygdala are particularly vulnerable (Burtscher et al., 2021, Wang Z. X. et al., 2022). Specific hippocampal and amygdalar subregions were changed in infant rats exposed to intermittent hypoxia, resulting in impaired memory, learning ability, and increased anxiety (Takenouchi et al., 2025). Similar outcomes have also been observed in rats exposed to high-altitude chronic hypoxia (Das et al., 2018). For HAs, greater cardiorespiratory fitness can effectively prevent volumetric decreases in hippocampus and amygdala from hypoxia (Wang Z. X. et al., 2022). However, morphological changes in the hippocampal and amygdalar subregions in long-term HAs have not been well-defined. In this study, a novel parcellation tool based on a probabilistic atlas derived from ultra-high-resolution *ex vivo* MRI data was applied to segment the hippocampus and amygdala (Iglesias et al., 2015, Saygin et al., 2017). We predicted that in comparison to the low-altitude control (LA) group, the HA group would show distinct patterns of volume changes in the hippocampal subfields and amygdalar nuclei, and specific structural alterations might link to neuropsychological impairments. Our findings reveal new morphological markers of high-altitude exposure in subcortical structures, providing factual support for the development of neuroimaging-based safety assessments for high-altitude adaptation and targeted health monitoring for high-altitude workers.

## 2 Materials and methods

### 2.1 Subjects

**FIGURE 1:**
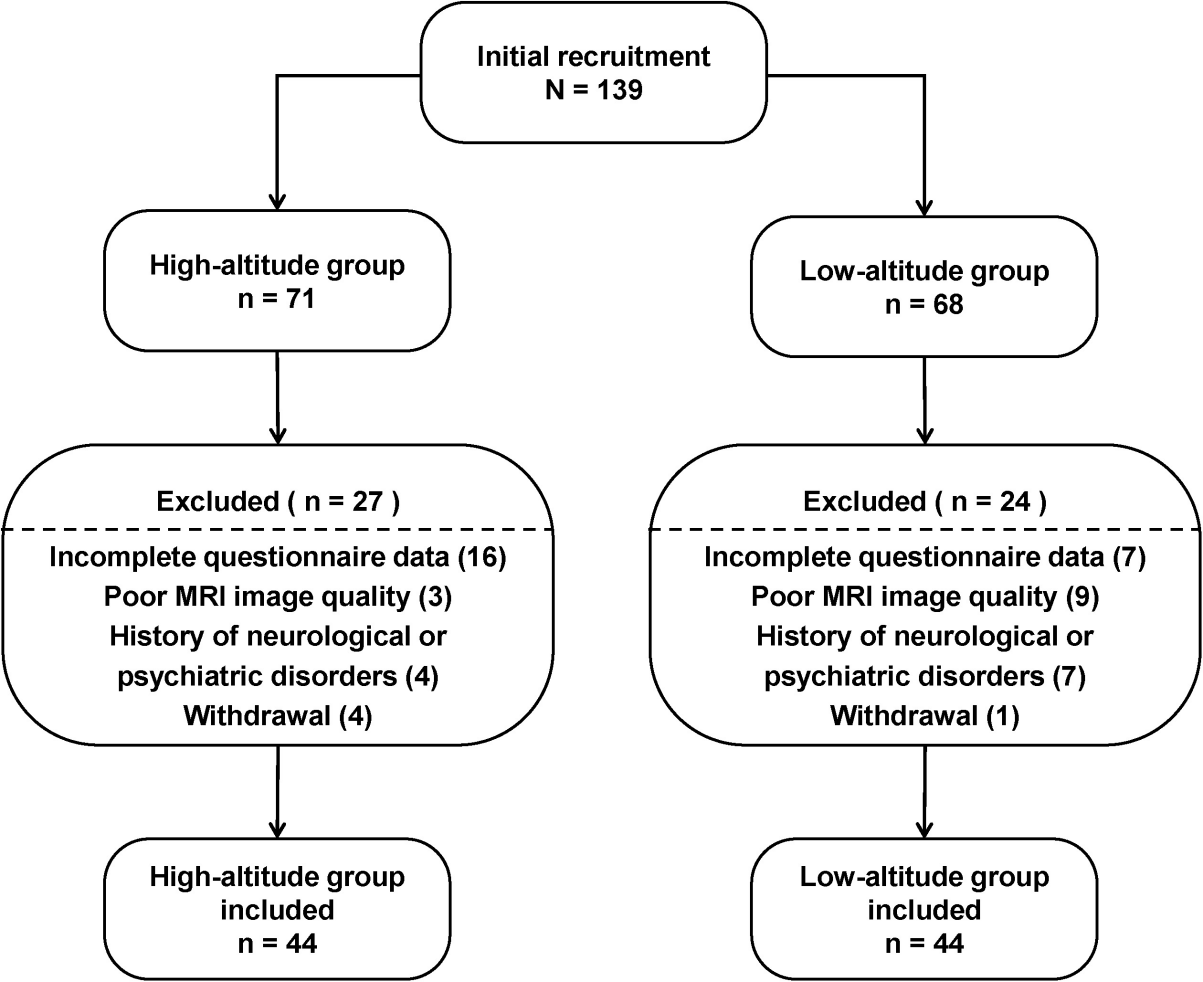
Participant recruitment of the study.

Our study divided participants into two groups: the HA group and the LA group, with 71 and 68 participants recruited from the outpatient clinic of the 945th Hospital of PLA, respectively. The inclusion criteria for the HA group were as follows: the participants had to be born and raised in low-altitude regions (below 500 meters), have migrated to the Tibetan Plateau (ranging from 3,000 to 4,000 meters) for long-term residence after adulthood, and have at least 2 years of continuous high-altitude exposure. The inclusion criterion for the LA group was that the participants had to be born and live permanently in low-altitude regions (below 500 meters), with no history of travel or residence in high-altitude regions. The exclusion criteria for both groups were a history of alcohol or substance use disorders, vascular risk factors, psychiatric disease, traumatic brain injury, neurological disease, or any contraindications to MRI. After screening, each group consisted of 44 matched male participants for the final analysis (Figure 1).

Due to the limited participant source and in order to control demographic variability and aging effects, all participants were right-handed Han Chinese males aged 20–30 years. All participants included in the study signed an informed consent form before the study started. The study protocol received approval from the Medical Ethics Committee of our hospital and was carried out in accordance with the Declaration of Helsinki principles.

### 2.2 Clinical and neuropsychological assessments

The duration of high-altitude residence for each HA participant was recorded using a standardized protocol. All participants completed structured interviews and underwent independent neuropsychological testing, which included the Self-Rating Depression Scale (SDS) to assess depressive symptoms, the Self-Rating Anxiety Scale (SAS) to evaluate anxiety state, the Insomnia Severity Index (ISI) to measure sleep disturbances, and the Positive and Negative Affect Schedule (PANAS) to determine affective state. For participants of the HA group, neuropsychological assessments and neuroimaging acquisition were conducted within 24 h after they returned to low-altitude areas.

### 2.3 MRI acquisition

A 1.5-Tesla uMR580 MRI scanner (United Imaging Healthcare, Shanghai, PRC) equipped with a 16-channel phase-array head coil was used to acquire three-dimensional high-resolution structural images. The participants were positioned in the supine position and were instructed to keep their heads completely still throughout the scan. Before high-resolution structural imaging was performed, routine clinical sequences, including T1-weighted, T2-weighted, and fluid-attenuated inversion recovery (FLAIR) MR images, were obtained to screen for structural abnormalities and exclude participants with organic brain lesions or significant white matter hyperintensities. The following magnetization-prepared rapid gradient echo (MPRAGE) acquisition parameters were used: repetition time (TR) = 8.4 ms; echo time (TE) = 3.4 ms; inversion time (TI) = 900 ms; flip angle = 10°; field of view (FOV) = 256 × 256 mm; slice thickness = 1 mm; number of slices = 192; and voxel size = 1 mm × 1 mm × 1 mm.

### 2.4 MRI analysis

We first verified that all images were not impacted by head movement. After quality control, the eligible images were processed via FreeSurfer software (version 7.2.0, Massachusetts General Hospital, Boston, MA, United States, http://surfer.nmr.mgh.harvard.edu). The post-processing stream has been described in detail in previous studies (Iglesias et al., 2015, Saygin et al., 2017, Fischl et al., 2004, Fischl et al., 2002). After the automated processing, the reconstructed cortical surfaces and subcortical segmentation were investigated to determine whether they followed gray matter boundaries and intensity borders, respectively, and if they were not aligned, each error was manually modified for the proper segmentation.

Hippocampal segmentation was performed based on a detailed subfield protocol (Iglesias et al., 2015), and includes the parasubiculum (PaS), presubiculum (PrS), subiculum (SUB), cornu ammonis (CA)1, CA3, CA4, granule cell and molecular layer of the dentate gyrus (GC-ML-DG), molecular layer (ML), hippocampal-amygdala transition area (HATA), fimbria, hippocampal tail (HT), and hippocampal fissure (HF). Additionally, the CA1, CA3, CA4, GC-ML-DG, ML, presubiculum, and subiculum were further segmented into head and body portions.

According to a well-established protocol (Saygin et al., 2017), the amygdala was segmented into 9 distinct nuclei: the anterior amygdaloid area (AAA), cortico-amygdala transition area (CAT), lateral nucleus (LA), basal nucleus (BA), paralaminar nucleus (PL), accessory basal nucleus (AB), medial nucleus (MA), central nucleus (CeA), and cortical nucleus (CoA).

### 2.5 Statistical analysis

All statistical analyses were performed using SPSS 22.0 software (IBM Inc., Armonk, New York, United States). We used independent samples *t*-tests to assess between-group differences in neuropsychological test scores and gray matter volumes. Where appropriate, the false discovery rate (FDR) correction was applied to between-group analyses that involved multiple comparisons, with an adjusted *p* < 0.05 considered statistically significant. Corrections were performed separately for the hippocampal subfields and the amygdalar nuclei.

For the HA group, correlations between the altered regional gray matter volume and neuropsychological scores were analyzed using Pearson correlation analysis or Spearman correlation analysis. The *p* level indicating statistical significance was set at < 0.05 without correction for multiple comparisons to assess potential trends.

## 3 Results

### 3.1 Participant characteristics

The demographic characteristics and neuropsychological test results are summarized in Table 1. The average duration of high-altitude residence in the HA group was 7.70 ± 3.84 (mean ± SD) years.

**TABLE 1:** Demographic features and neuropsychological performance.

Characteristics	HA (*n* = 44)	LA (*n* = 44)	*t*	*P*
Age (years)	25.70 ± 3.84	24.59 ± 2.73	1.57	0.12
Duration of high-altitude residence (years)	7.70 ± 3.84	–	–	–
SDS score	54.16 ± 11.44	34.95 ± 6.18	9.80	< 0.001**
SAS score	42.50 ± 9.71	40.55 ± 4.42	1.22	0.23
ISI score	10.00 ± 4.31	6.75 ± 3.66	3.81	< 0.001**
Positive affect score	34.43 ± 8.62	34.07 ± 6.66	0.22	0.83
Negative affect score	19.16 ± 5.92	19.18 ± 6.16	−0.02	0.99

HA, high-altitude migrant; LA, low-altitude control; SDS, self-rating depression scale; SAS, self-rating anxiety scale; ISI, insomnia severity index. Continuous variables are expressed as the means ± SDs. ** *p* < 0.001.

Between-group comparisons revealed significant differences in neuropsychological measures: SDS and ISI scores were higher in the HA group than in the LA group (both *p* < 0.05). The two groups did not differ significantly in age, state SAS scores, or positive/negative affect scores (all *p* > 0.05).

### 3.2 Gray matter volume comparisons

Volumetric comparisons between groups are presented in Table 2. Independent samples *t*-tests revealed that, compared with the LA group, the HA group presented significantly reduced total subcortical gray matter volume (*t* = −2.04, *p* = 0.044). No significant differences in volume in the left cerebral cortex, right cerebral cortex, left cerebellar cortex, right cerebellar cortex, or total gray matter volume were found between the groups (all *p* > 0.05).

**TABLE 2:** Comparison of brain volume between the HA group and the LA group.

Volumes (mm^3^)	HA	LA	*t*	*P*
Left cerebrum cortex	260278.79 ± 20006.94	262696.21 ± 23005.29	−0.53	0.600
Right cerebrum cortex	260821.88 ± 19911.06	264423.41 ± 23067.72	−0.78	0.435
Left cerebellum cortex	56656.63 ± 4578.18	56143.53 ± 5897.17	0.46	0.650
Right cerebellum cortex	57474.16 ± 4263.15	56746.55 ± 6258.2	0.64	0.526
Subcortical gray matter	61659.32 ± 3756.69	63733.09 ± 5598.29	−2.04	0.044 *
Total gray matter	697056.15 ± 44893.81	703703.55 ± 57862.48	−0.60	0.549

HA, high-altitude migrant; LA, low-altitude control. Continuous variables are expressed as the means ± SDs. *p < 0.05.

### 3.3 Hippocampal subfield volumetric differences

**FIGURE 2:**
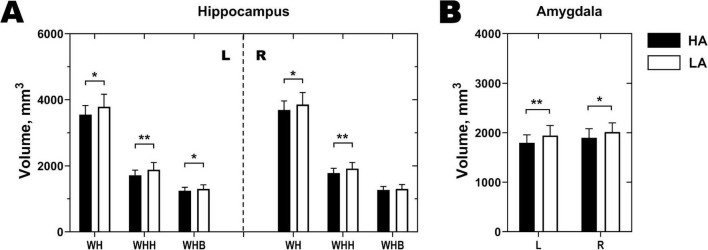
Differential volumes in the hippocampus and amygdala between the HA group and the LA group. HA, high-altitude migrant; LA, low-altitude control; WH, whole hippocampus; WHH, whole hippocampal head; WHB, whole hippocampal body; L, left; R, right. *p< 0.05. **p < 0.001.

The whole-hippocampus structural analysis revealed that the HA group had significantly smaller bilateral whole hippocampus volume, bilateral whole hippocampal head volume, and left whole hippocampal body volume than the LA group (*p* < 0.05; Figure 2A).

**FIGURE 3:**
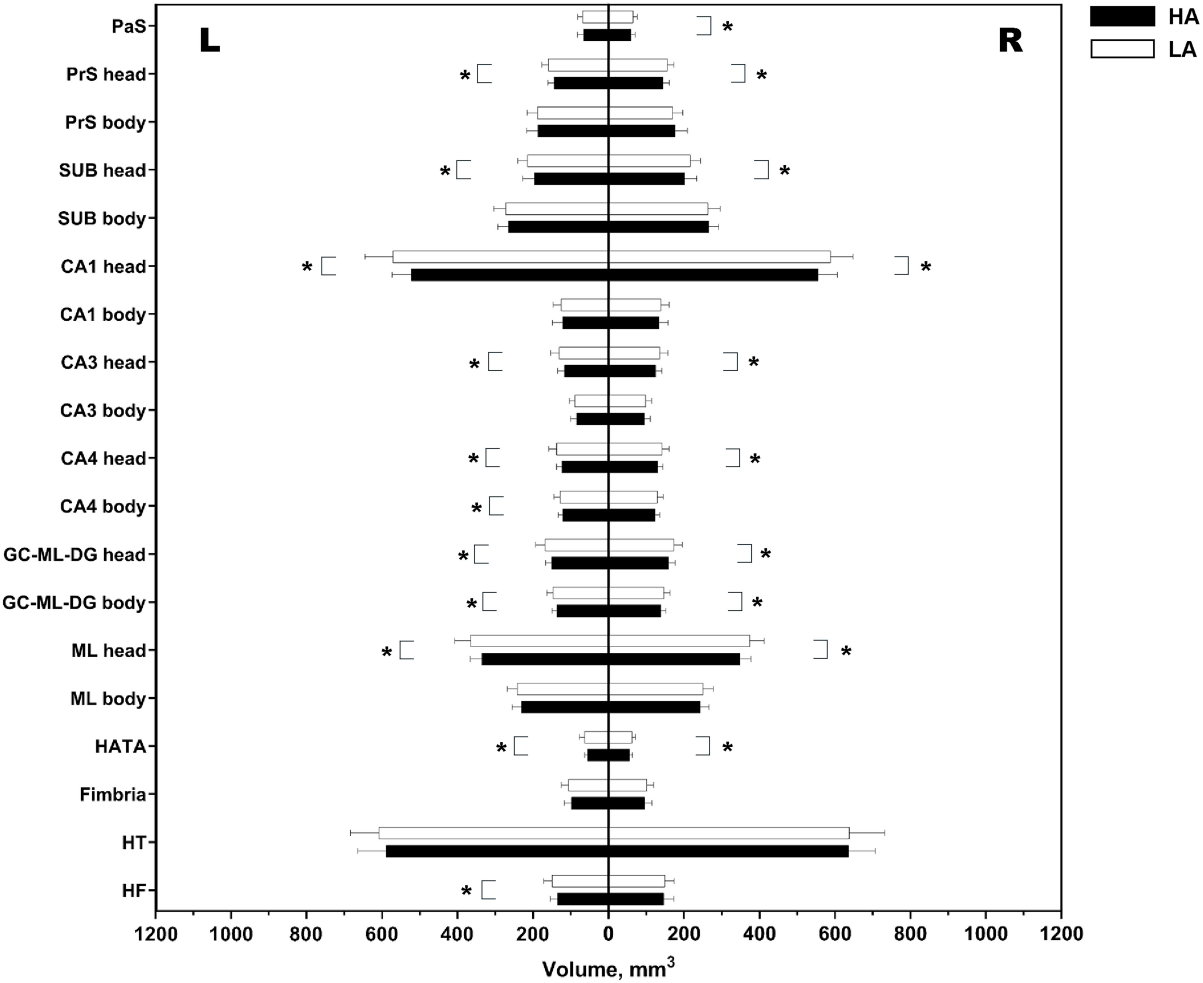
Differential volumes of hippocampal subfields between the HA group and the LA group. HA, high-altitude migrant; LA, low-altitude control; PaS, parasubiculum; PrS, presubiculum; SUB, subiculum; CA, cornu ammonis; GC-ML-DG, granule cell and molecular layer of the dentate gyrus; ML, molecular layer; HATA, hippocampal-amygdala transition area; HT, hippocampal tail; HF, hippocampal fissure; L, left; R, right. *Significant FDR-corrected *p* < 0.05.

More detailed subfield analysis revealed widespread volumetric reductions in the HA group after multiple comparison correction (FDR-corrected *p* < 0.05, Figure 3). Volumetric decreased subregions included the head portions of the bilateral SUB, PrS, CA1, CA3, CA4, GC-ML-DG, and ML, as well as the body segments of the bilateral GC-ML-DG and left CA4. In addition, significant reductions in the left HF, right PaS, and bilateral HATA in the HA group were observed.

### 3.4 Amygdalar nuclei volumetric differences

A comparison of the whole-amygdala measurements revealed that, compared with the LA group, the HA group presented a decreased bilateral amygdala volume (*p* < 0.05; Figure 2B).

**FIGURE 4:**
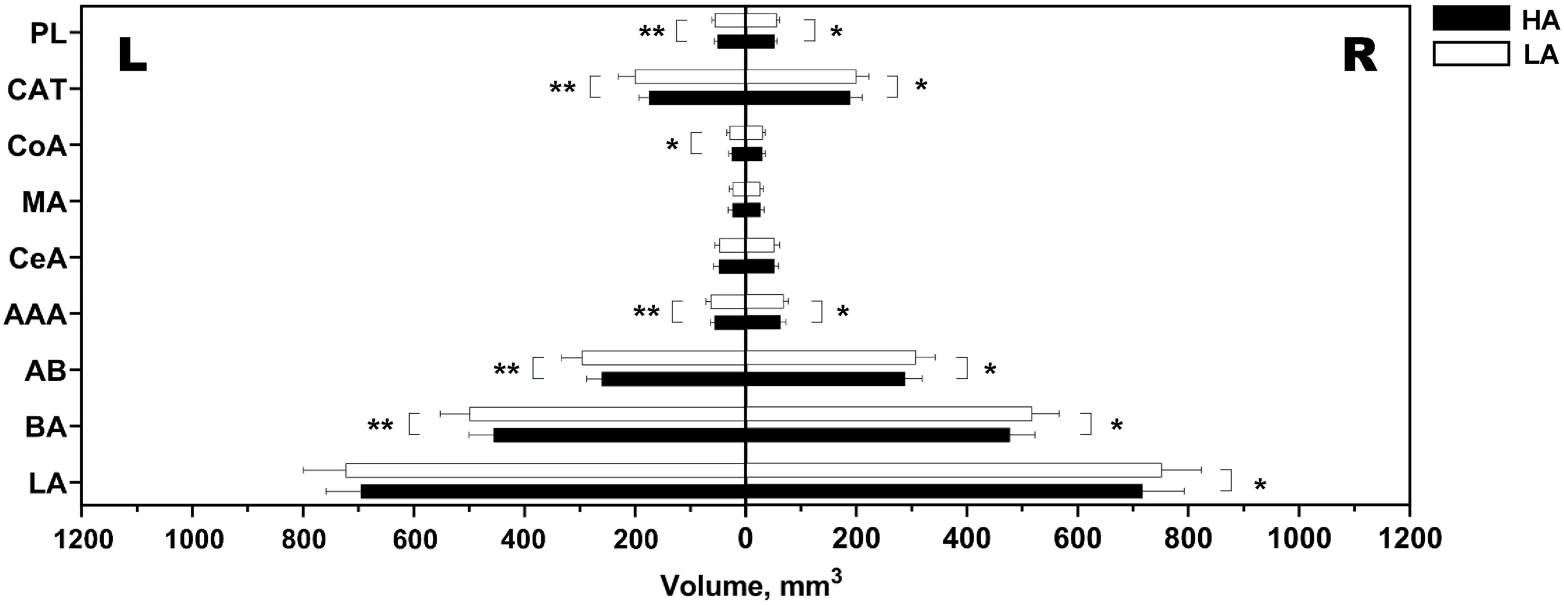
Differential volumes of amygdalar nuclei between the HA group and the LA group. HA, high-altitude migrant; LA, low-altitude control; PL, paralaminar nucleus; CAT, cortico-amygdala transition area; CoA, cortical nucleus; MA, medial nucleus; CeA, central nucleus; AAA, anterior amygdaloid area; AB, accessory basal nucleus; BA, basal nucleus; LA, lateral nucleus; L, left; R, right. *Significant FDR-corrected *p* < 0.05. **Significant FDR-corrected *p* < 0.001.

More detailed subregional analyses revealed specific amygdalar nuclei showing volume reductions in the HA group, including the right LA, bilateral BA, bilateral AB, bilateral AAA, left CoA, bilateral CAT, and bilateral PL, all of which survived FDR-correction at *p* < 0.05 (Figure 4).

### 3.5 Correlation analysis

**FIGURE 5:**
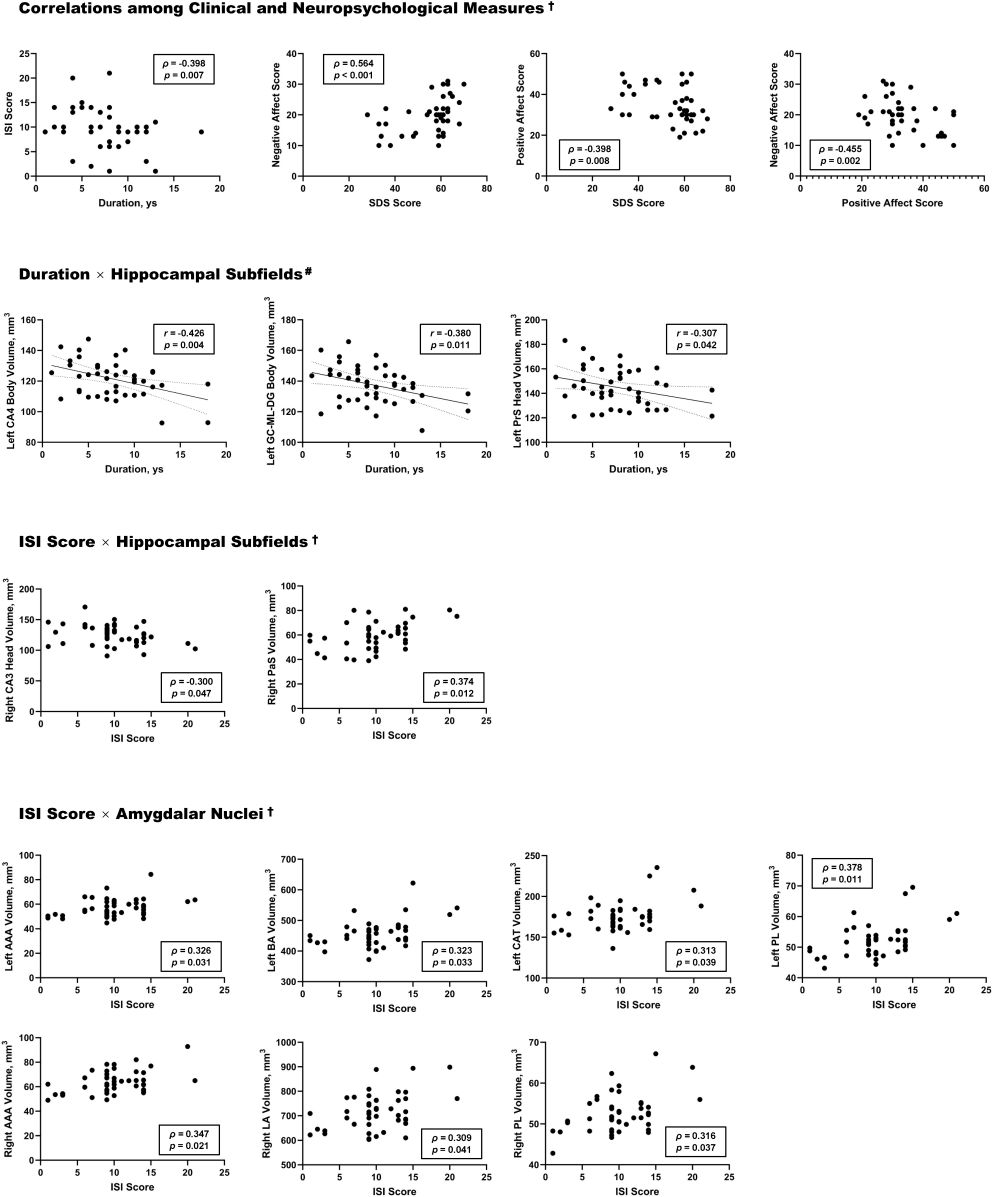
Correlations among clinical measures, neuropsychological test scores, and volumes of hippocampal subfields and amygdalar nuclei in the HA group. HA, high-altitude migrant; ISI, Insomnia Severity Index; SDS, Self-Rating Depression Scale; CA, cornu ammonis; GC-ML-DG, granule cell and molecular layer of dentate gyrus; PrS, presubiculum; PaS, parasubiculum; AAA, anterior amygdaloid area; BA, basal nucleus; CAT, cortico-amygdala transition area; PL, paralaminar nucleus; LA, lateral nucleus. † Spearman correlation (applied for non-normally distributed variables). # Pearson correlation (applied for normally distributed variables).

Several significant associations among clinical, neuropsychological, and neuroanatomical measurements in the HA group were identified (Figure 5). A longer duration of high-altitude residence was associated with lower insomnia severity (*r* = −0.40, *p* = 0.0007, ISI score). SDS score was negatively associated with positive affect score (*r* = −0.40, *p* = 0.0007) and positively associated with negative affect score (*r* = −0.40, *p* = 0.0007). Gray matter volume in multiple areas was correlated with ISI scores, including robust positive associations in the right PaS (*r* = 0.34, *p* = 0.012), and multiple amygdala subnuclei (including the left BA, bilateral AAA, right LA, left CAT, and bilateral PL; all *p* < 0.05). In contrast, the right CA3 head was negatively correlated with the ISI score (*r* = −0.30, *p* = 0.047).

In addition, longer high-altitude exposure was linked to reduced volume in the left PrS head (*r* = −0.31, *p* = 0.042), left GC-ML-DG body (*r* = −0.38, *p* = 0.011), and left CA4 body (*r* = −0.43, *p* = 0.004).

## 4 Discussion

The current study investigated structural alterations in male participants in early adulthood who migrated from low-altitude areas to high-altitude regions for long-term residence, compared with matched low-altitude resident controls. Volumetric analyses revealed essentially preserved global gray matter volumes in both hemispheres and the cerebellum, but significant reductions in whole hippocampal and amygdalar volumes, as well as widespread reductions in hippocampal and amygdalar subregional volumes, in the HA group. These morphological changes were differentially correlated with both neuropsychological measures and duration of high-altitude exposure.

The unique neuroanatomical alterations in HAs revealed in our study are in line with a recent cross-sectional study (Feng et al., 2023) and provide new evidence of the impact of high-altitude exposure on limbic system morphology. The hippocampus and the striatum are more sensitive to oxygen deprivation than cortical areas, and oxidative stress is time-dependent (Maiti et al., 2006, Wang X. et al., 2022). In addition, the initial adaptation to acute hypoxia involves increased cerebral blood flow (Wang X. et al., 2022), whereas more chronic responses may involve paradoxical microvascular pruning, angiogenesis, and neovascularization (Koester-Hegmann et al., 2018; Wang X. et al., 2022). Distinct patterns were shown in total cortical volume and total subcortical volume in HAs and may indicate the remarkable structural flexibility at the surface level of the brain and the structural vulnerability of deeper brain tissue. This could be a result of hypoxia-related cerebrovascular remodeling processes: oxygen might be preferentially delivered to superficial regions rather than deep structures due to distinct vascular architecture and blood perfusion in different layers of the brain (Pfefferbaum et al., 2010, Farahani et al., 2025, Konan et al., 2018).

One of the prominent characteristics of the volumetric changes in the hippocampal and amygdalar subregions is almost symmetry, suggesting that high-altitude exposure affects the limbic structures integrity in a systematic way as opposed to randomly or laterally. Another significant characteristic of these changes is regional specificity. Prolonged oxygen deprivation can set off oxidative stress, neuroinflammation, reduction in neurotrophic factor expression, apoptosis, etc. (Pialoux and Mounier, 2012, Gong et al., 2020, Xie and Yung, 2012, Shchelchkova et al., 2020, Wang X. et al., 2022), all of which together promote neuronal degeneration (Peers et al., 2009, Merelli et al., 2018, Wang X. et al., 2022) and structural reorganization (Chen et al., 2016, Xie and Yung, 2012). Moreover, hypoxia-induced vascular changes, such as vascular endothelial growth factor (VEGF)-mediated increases in blood-brain barrier permeability (Yeh et al., 2007, Jamieson et al., 2022), may be enhanced by hypoxia-inducible factor (HIF)-1 (Shaharuddin et al., 2023). These hemodynamic alterations appear to selectively induce neuronal apoptosis and necroptosis (Koester-Hegmann et al., 2018, Huan et al., 2023), which might explain the regionally specific vulnerability observed in our subregional analyses.

The primary manifestation of the regionally specific vulnerability of hippocampal subfields in HAs is that volume loss in the head portions was more significant and extensive than that in the body portions, and the tail portions did not show any significant volumetric changes. Because the head, body, and tail of the hippocampus are structurally and functionally connected to different cerebral regions, their functional relevance is considered to be dissociable (Canada et al., 2020). The bilateral volume decreases in the CA subfields (CA1, CA3, and CA4) and the GC-ML-DG region suggest possible impairments in the trisynaptic loop, a key circuit for learning and memory encoding (Shinohara and Kohara, 2023). The significant volume reduction in the HATA, which connects the hippocampus and amygdala, may also be a structural marker that emotional memory processing is altered under high-altitude conditions. These subregional specific vulnerabilities might indicate various levels of disruption in hippocampal processing that involves different neural networks.

According to the classification proposed by Sah et al. (2003), the amygdala nuclei are divided into four groups: the deep or basolateral nuclei, the superficial or cortical-like nuclei, the centromedial nuclei, and other amygdaloid nuclei. In our study, remarkable volumetric reductions across multiple amygdalar nuclei were detected in HAs except for the centromedial group (i.e., the CeA and MA), further demonstrating selective impacts of high-altitude exposure on the limbic system. The CeA serves as the primary integration center in the amygdala circuitry for receiving emotionally related information from sensory areas, projecting to effector systems that regulate physiological and behavioral responses, and coordinating a variety of adaptive behaviors, including defensive responses and feeding-seeking responses (Fadok et al., 2018, Yeh et al., 2024). The MA, a crucial hub implicated in the control of social and defensive/stress-relevant behaviors, integrates both internal state and external emotional inputs for social and emotional context adaptations (Petrulis, 2020). The selective preservation of centromedial nuclei seems to be the outcome of self-adaptive regulation, which enables other amygdala nuclei to undergo volume reduction preferentially under high-altitude conditions.

Depression is a common mental disorder among high-altitude populations (Ma et al., 2024, Kious et al., 2018, Basualdo-Melendez et al., 2022). This phenomenon was also observed in our study. Depression is almost certainly caused by various interrelated neurobiological mechanisms, such as the dysregulation of neurotransmitters in the serotonin and dopamine systems (Nutt, 2008), structural atrophy in the limbic circuits that regulate mood (Banasr et al., 2011), and impaired function of the HPA axis (Menke, 2024). The correlation between SDS score and positive/negative affect score indicates that anhedonia and emotional dysregulation may play a crucial role in high-altitude depression. Furthermore, sleep disturbances may exacerbate neurobiological abnormalities by synergistically impairing memory function and emotional processing (Killgore, 2010). However, insomnia symptoms in HAs were reduced over time, implying that prolonged exposure might activate endogenous adaptive processes that can partially restore functional homeostasis.

The positive association between insomnia severity and specific amygdalar subregion volume appears to be paradoxical. The possible explanation is that, during the adaptive process, HAs may suffer from excessive emotional processing triggered by amygdalar hyper-reactivity, which in turn disturbs sleep regulation. Furthermore, compared with long-term HAs, HAs with short exposure time usually have poorer sleep quality and relatively intact amygdala structures. For the hippocampus, specific subfields exhibit time-dependent volume reductions or are associated with insomnia. This suggests that these microstructures may play distinct functional roles in the adaptation to high-altitude exposure. It should be noted that volume decrease does not consequently indicate maladaptive neurodegeneration or functional impairment. Instead, it might also represent strategic neuroplastic reorganization under long-term hypoxia: certain subcircuits undergo volume reduction to prune less essential connections, while others preserve their structure to maintain essential functions.

Several limitations should be acknowledged. First, this was a cross-sectional study, and the variation in the duration of high-altitude exposure within the HA group is an uncontrollable source of heterogeneity. Although demographic matching decreases the probability of pre-existing differences between groups, the absence of pre-exposure neuroimaging data and duration-stratified analysis (e.g., short-term vs. long-term exposure) implies that we cannot completely rule out baseline variations and the temporal progression of neuroplastic changes. Second, our sampling pool exclusively recruited male Han Chinese participants because the majority of HAs are of Han Chinese ethnicity, and males predominate in high-altitude occupational categories in China. Although the homogeneity of our samples is beneficial for reducing confounding variables, such as hormonal effects on cerebral blood flow regulation that differ by sex, and hypoxia adaptation that differ by ethnicity, this design limitation restricts the general applicability of the findings to broader populations. Additionally, male-dominated occupational factors might introduce cohort-specific biases independent of altitude effects. Third, our exclusive reliance on structural MRI, without including functional or metabolic imaging data, limits our understanding of the functional network adaptations that occur along with structural alteration. Fourth, a possible confounding factor is the wide altitude range within the HA group. The dose-response relationships may be hidden as a result. Fifth, the education level of participants was not collected in our study. The homogeneity of our sample (e.g., all participants were young adult males) may partially alleviate educational variability, but it is impossible to completely rule out the confounding influence of unmeasured variations in educational attainment data on our findings.

In conclusion, our study revealed complex patterns of neuroanatomical vulnerability and adaptability in young adult male Han Chinese HAs. Subregion-level analysis demonstrated symmetrical and distinct vulnerabilities in the hippocampal subfields and amygdalar subnuclei in HAs. Long-term high-altitude residence was correlated with reduced insomnia symptomatology in HAs, indicating the emergence of neurobiological adaptation over time. These findings advance our understanding of the consequences of chronic hypoxia exposure as well as the remarkable brain capacity for structural plasticity in extreme environments. Our findings further highlight the importance of monitoring brain health and implementing protective strategies for healthy management in HAs.

## Data Availability

The datasets presented in this article are not readily available because privacy concerns or ethical restrictions of the data. Requests to access the datasets should be directed to Jian Zhou, 363768420@qq.com.

## References

[B1] Banasr M. Dwyer J. M. Duman R. S. (2011). Cell atrophy and loss in depression: Reversal by antidepressant treatment. *Curr. Opin. Cell Biol.* 23 730–737. 10.1016/j.ceb.2011.09.002 21996102 PMC3259683

[B2] Basualdo-Melendez G. W. Hernandez-Vasquez A. Baron-Lozada F. A. Vargas-Fernandez R. (2022). Prevalence of depression and depressive symptoms at high altitudes: A systematic review and meta-analysis. *J. Affect. Disord.* 317 388–396. 36055536 10.1016/j.jad.2022.08.079

[B3] Burtscher J. Mallet R. T. Burtscher M. Millet G. P. (2021). Hypoxia and brain aging: Neurodegeneration or neuroprotection? *Ageing Res. Rev.* 68 101343. 10.1016/j.arr.2021.101343 33862277

[B4] Canada K. L. Botdorf M. Riggins T. (2020). Longitudinal development of hippocampal subregions from early- to mid-childhood. *Hippocampus* 30 1098–1111. 10.1002/hipo.23218 32497411 PMC8500647

[B5] Chen J. Li J. Han Q. Lin J. Yang T. Chen Z. et al. (2016). Long-term acclimatization to high-altitude hypoxia modifies interhemispheric functional and structural connectivity in the adult brain. *Brain Behav.* 6:e00512. 10.1002/brb3.512 27688941 PMC5036434

[B6] Das D. Biswal S. Barhwal K. K. Chaurasia O. P. Hota S. K. (2018). Kaempferol inhibits extra-synaptic nmdar-mediated downregulation of trkbeta in rat hippocampus during hypoxia. *Neuroscience* 392 77–91. 10.1016/j.neuroscience.2018.09.018 30266684

[B7] Fadok J. P. Markovic M. Tovote P. Luthi A. (2018). New perspectives on central amygdala function. *Curr. Opin. Neurobiol.* 49 141–147. 10.1016/j.conb.2018.02.009 29522976

[B8] Farahani A. Liu Z. Q. Ceballos E. G. Hansen J. Y. Wennberg K. Zeighami Y. et al. (2025). Mapping cerebral blood perfusion and its links to multi-scale brain organization across the human lifespan. *PLoS Biol*. 23:e3003277. 10.1371/journal.pbio.3003277 40729400 PMC12324687

[B9] Feng J. Men W. Yu X. Liu W. Zhang S. Liu J. et al. (2023). High-altitude exposure duration dependent global and regional gray matter volume decrease in healthy immigrants: A cross-sectional study. *Acta Radiol.* 64 751–759. 10.1177/02841851221091674 35369766

[B10] Fischl B. Salat D. H. Busa E. Albert M. Dieterich M. Haselgrove C. et al. (2002). Whole brain segmentation: Automated labeling of neuroanatomical structures in the human brain. *Neuron* 33 341–355. 10.1016/s0896-6273(02)00569-x 11832223

[B11] Fischl B. van der Kouwe A. Destrieux C. Halgren E. Ségonne F. Salat D. H. et al. (2004). Automatically parcellating the human cerebral cortex. *Cereb. Cortex* 14 11–22. 10.1093/cercor/bhg087 14654453

[B12] Frank F. Kaltseis K. Filippi V. Broessner G. (2022). Hypoxia-related mechanisms inducing acute mountain sickness and migraine. *Front. Physiol.* 13:994469. 10.3389/fphys.2022.994469 36148300 PMC9485719

[B13] Gao X. Zhang Z. Li X. Li C. Hao J. Luo Y. et al. (2020). Macitentan attenuates chronic mountain sickness in rats by regulating arginine and purine metabolism. *J. Proteome Res.* 19 3302–3314. 10.1021/acs.jproteome.0c00219 32640793

[B14] Gong L.-J. Wang X.-Y. Gu W.-Y. Wu X. (2020). Pinocembrin ameliorates intermittent hypoxia-induced neuroinflammation through Bnip3-dependent mitophagy in a murine model of sleep apnea. *J. Neuroinflammation* 17:337. 10.1186/s12974-020-02014-w 33176803 PMC7656728

[B15] Huan Y. Quan H. Jia B. Hao G. Shi Z. Zhao T. et al. (2023). High-altitude cerebral hypoxia promotes mitochondrial dysfunction and apoptosis of mouse neurons. *Front. Mol. Neurosci.* 16:1216947. 10.3389/fnmol.2023.1216947 37501726 PMC10370763

[B16] Iglesias J. E. Augustinack J. C. Nguyen K. Player C. M. Player A. Wright M. et al. (2015). A computational atlas of the hippocampal formation using ex vivo, ultra-high resolution MRI: Application to adaptive segmentation of in vivo MRI. *Neuroimage* 115 117–137. 10.1016/j.neuroimage.2015.04.042 25936807 PMC4461537

[B17] Jamieson J. J. Lin Y. Malloy N. Soto D. Searson P. C. Gerecht S. (2022). Hypoxia-induced blood-brain barrier dysfunction is prevented by pericyte-conditioned media via attenuated actomyosin contractility and claudin-5 stabilization. *FASEB J.* 36:e22331. 10.1096/fj.202200010RR 35476363 PMC9060394

[B18] Killgore W. D. (2010). Effects of sleep deprivation on cognition. *Prog. Brain Res.* 185 105–129. 10.1016/B978-0-444-53702-7.00007-5 21075236

[B19] Kious B. M. Kondo D. G. Renshaw P. F. (2018). Living high and feeling low: Altitude, suicide, and depression. *Harv. Rev. Psychiatry* 26 43–56. 10.1097/HRP.0000000000000158 29517615

[B20] Koester-Hegmann C. Bengoetxea H. Kosenkov D. Thiersch M. Haider T. Gassmann M. et al. (2018). High-altitude cognitive impairment is prevented by enriched environment including exercise via VEGF signaling. *Front. Cell. Neurosci.* 12:532. 10.3389/fncel.2018.00532 30687018 PMC6335396

[B21] Konan L. M. Reddy V. Mesfin F. B. (2018). “Neuroanatomy, Cerebral Blood Supply,” in *StatPearls*, Treasure Island, FL: StatPearls Publishing. 30335330

[B22] Li Y. Wang Y. (2022). Effects of long-term exposure to high altitude hypoxia on cognitive function and its mechanism: A narrative review. *Brain Sci*. 12:808. 10.3390/brainsci12060808 35741693 PMC9221409

[B23] Liu N. Feng L. Chai S. Li H. He Y. Guo Y. et al. (2024). A diffusion tensor imaging-based multidimensional study of brain structural changes after long-term high-altitude exposure and their relationships with cognitive function. *Front. Physiol.* 15:1487953. 10.3389/fphys.2024.1487953 39605859 PMC11599258

[B24] Ma Q. Jiang W. Zhao Q. Xia X. Fang R. (2024). Relationships between altitude and depressive symptoms among middle-aged and older adults in China: A longitudinal study from the China health and retirement longitudinal study. *Front. Psychiatry* 15:1436541. 10.3389/fpsyt.2024.1436541 39473918 PMC11518746

[B25] Maiti P. Singh S. B. Sharma A. K. Muthuraju S. Banerjee P. K. Ilavazhagan G. (2006). Hypobaric hypoxia induces oxidative stress in rat brain. *Neurochem. Int.* 49 709–716. 10.1016/j.neuint.2006.06.002 16911847

[B26] Menke A. (2024). The HPA axis as target for depression. *Curr. Neuropharmacol.* 22 904–915. 10.2174/1570159X21666230811141557 37581323 PMC10845091

[B27] Merelli A. Rodríguez J. C. G. Folch J. Regueiro M. R. Camins A. Lazarowski A. (2018). Understanding the role of hypoxia inducible factor during neurodegeneration for new therapeutics opportunities. *Curr. Neuropharmacol.* 16 1484–1498. 10.2174/1570159X16666180110130253 29318974 PMC6295932

[B28] Nutt D. J. (2008). Relationship of neurotransmitters to the symptoms of major depressive disorder. *J. Clin. Psychiatry* 69(Suppl. E1), 4–7. 18494537

[B29] Peers C. Dallas M. L. Boycott H. E. Scragg J. L. Pearson H. A. Boyle J. P. (2009). Hypoxia and neurodegeneration. *Ann. N. Y. Acad. Sci.* 1177 169–177. 10.1111/j.1749-6632.2009.05026.x 19845619

[B30] Petrulis A. (2020). Structure and function of the medial amygdala. *Handb. Behav. Neurosci.* 26 39–61. 10.1016/B978-0-12-815134-1.00002-7

[B31] Pfefferbaum A. Chanraud S. Pitel A.-L. Shankaranarayanan A. Alsop D. C. Rohlfing T. et al. (2010). Volumetric cerebral perfusion imaging in healthy adults: Regional distribution, laterality, and repeatability of pulsed continuous arterial spin labeling (Pcasl). *Psychiatry Res. Neuroimaging* 182 266–273. 10.1016/j.pscychresns.2010.02.010 20488671 PMC2914847

[B32] Pialoux V. Mounier R. (2012). Hypoxia-induced oxidative stress in health disorders. *Oxid. Med. Cell. Longev*. 2012:940121. 10.1155/2012/940121 23304260 PMC3530865

[B33] Quaresma M. Souza W. Lemos V. Caris A. Santos R. V. T. D. (2020). The possible importance of glutamine supplementation to mood and cognition in hypoxia from high altitude. *Nutrients* 12:3627. 10.3390/nu12123627 33255790 PMC7760805

[B34] Sah P. Faber E. S. Lopez De Armentia M. Power J. (2003). The amygdaloid complex: Anatomy and physiology. *Physiol. Rev.* 83 803–834. 10.1152/physrev.00002.2003 12843409

[B35] Saygin Z. M. Kliemann D. Iglesias J. E. van der Kouwe A. J. W. Boyd E. Reuter M. et al. (2017). High-resolution magnetic resonance imaging reveals nuclei of the human amygdala: Manual segmentation to automatic atlas. *Neuroimage* 155 370–382. 10.1016/j.neuroimage.2017.04.046 28479476 PMC5557007

[B36] Shaharuddin S. Nik Abd Rahman N. M. A. Masarudin M. J. Alamassi M. N. Ahmad Saad F. F. (2023). HIF-1 sensor in detecting hypoxia tolerance at high altitude. *Aerosp. Med. Hum. Perform*. 94 485–487. 10.3357/AMHP.6166.2023 37194178

[B37] Shchelchkova N. A. Kokaya A. A. Bezhenar’ V. F. Rozhdestvenskaya O. V. Mamedova M. A. Mishchenko T. A. et al. (2020). The role of brain-derived neurotrophic factor and glial cell line-derived neurotrophic factor in chronic fetal oxygen deprivation. *Sovrem. Tekhnologii Med*. 12 25–31. 10.17691/stm2020.12.1.03 34513034 PMC8353703

[B38] Shinohara Y. Kohara K. (2023). Projections of hippocampal Ca2 pyramidal neurons: Distinct innervation patterns of Ca2 compared to Ca3 in rodents. *Hippocampus* 33 691–699. 10.1002/hipo.23519 36855258

[B39] Takenouchi Y. Hosomichi J. Angkanawaraphan K. Maeda H. Hong H. Changsiripun C. et al. (2025). Sex- and subregion-specific vulnerability of the hippocampus and amygdala to intermittent hypoxia in relation to learning/memory function and anxiety tendencies of infant rats. *Sleep Breath.* 29:209. 10.1007/s11325-025-03382-4 40490588 PMC12148965

[B40] Wang X. Cui L. Ji X. (2022). Cognitive impairment caused by hypoxia: From clinical evidences to molecular mechanisms. *Metab. Brain Dis.* 37 51–66. 10.1007/s11011-021-00796-3 34618295

[B41] Wang Z. X. Su R. Li H. Dang P. Zeng T. A. Chen D. M. et al. (2022). Changes in hippocampus and amygdala volume with hypoxic stress related to cardiorespiratory fitness under a high-altitude environment. *Brain Sci*. 12:359. 10.3390/brainsci12030359 35326315 PMC8946638

[B42] Xie H. Yung W.-H. (2012). Chronic intermittent hypoxia-induced deficits in synaptic plasticity and neurocognitive functions: A role for brain-derived neurotrophic factor. *Acta Pharmacol. Sin*. 33 5–10. 10.1038/aps.2011.184 22212429 PMC4010262

[B43] Yeh L. F. Zuo S. Liu P. W. (2024). Molecular diversity and functional dynamics in the central amygdala. *Front. Mol. Neurosci.* 17:1364268. 10.3389/fnmol.2024.1364268 38419794 PMC10899328

[B44] Yeh W. L. Lu D. Y. Lin C. J. Liou H. C. Fu W. M. (2007). Inhibition of hypoxia-induced increase of blood-brain barrier permeability by YC-1 through the antagonism of HIF-1alpha accumulation and VEGF expression. *Mol. Pharmacol.* 72 440–449. 10.1124/mol.107.036418 17513385

[B45] Zhang X. Xie W. Du W. Liu Y. Lin J. Yin W. et al. (2023). Consistent differences in brain structure and functional connectivity in high-altitude native Tibetans and immigrants. *Brain Imaging Behav*. 17 271–281. 10.1007/s11682-023-00759-5 36694086

